# Feeding critically ill patients the right ‘whey’: thinking outside of the box. A personal view

**DOI:** 10.1186/s13613-015-0051-2

**Published:** 2015-05-28

**Authors:** Paul E Marik

**Affiliations:** Division of Pulmonary and Critical Care Medicine, Eastern Virginia Medical School, 825 Fairfax Av, Suite 410, Norfolk, VA 23507 USA

**Keywords:** Nutrition, Whey, Muscle mass, Muscle atrophy, Intermittent feeding, Continuous feeding, Metabolic function, Insulin, Incretin, MTOR, Autophagy, Ubiquitin–proteasome complex

## Abstract

Atrophy of skeletal muscle mass is an almost universal problem in survivors of critical illness and is associated with significant short- and long-term morbidity. Contrary to common practice, the provision of protein/amino acids as a continuous infusion significantly limits protein synthesis whereas intermittent feeding maximally stimulates skeletal muscle synthesis. Furthermore, whey-based protein (high in leucine) increases muscle synthesis compared to soy or casein-based protein. In addition to its adverse effects on skeletal muscle synthesis, continuous feeding is unphysiological and has adverse effects on glucose and lipid metabolism and gastrointestinal function. I propose that critically ill patients’ be fed intermittently with a whey-based formula and that such an approach is likely to be associated with better glycemic control, less hepatic steatosis and greater preservation of muscle mass. This paper provides the scientific basis for my approach to intermittent feeding of critically ill patients.

## Review

Survivors of critical illness suffer from marked muscle wasting which may take years to recover. The loss of muscle mass is associated with muscle weakness, prolonged mechanical ventilatory support, fatigue and delayed recovery [1–3]. This disorder is known as critical illness myopathy (CIM) or intensive care unit-acquired weakness (ICUAW) [1–3]. CIM is characterized by a diffuse non-necrotizing myopathy accompanied by fiber atrophy, fatty degeneration of muscle fibers and fibrosis [4]. Multiple factors are likely to play a role in inducing CIM including muscle inactivity, inflammation, cellular energy stress, corticosteroids, hyperglycemia, neuromuscular blocking agents and inadequate nutritional support [2, 4]. CIM is exceedingly common in ICU survivors, being reported in up to 46 % of cases [5]. Herridge et al. followed 109 survivors of ARDS for up to 5 years after discharge from the ICU [6, 7]. All patients reported poor functional status with proximal weakness and fatigue at discharge. At 1 year, the distance walked in 6 minutes was 66 % of predicted which increased to 76 % of predicted at 5 years [7].

### Muscle breakdown during acute illness

**Fig. 1:**
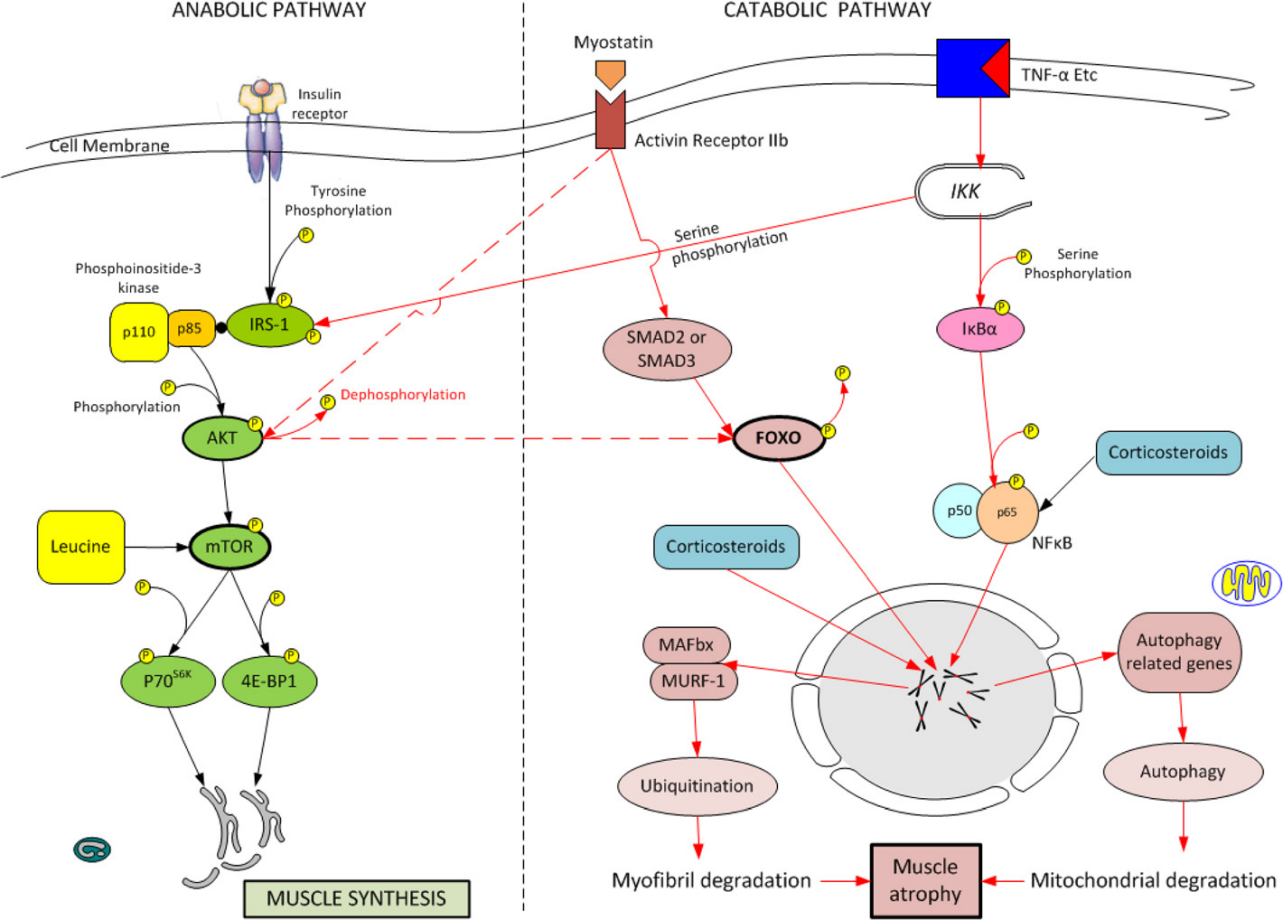
A simplified overview of the anabolic and catabolic pathways in skeletal muscle.AKT= protein kinase b;FOXO-1=forkhead box class O-1; IRS-1=insulin receptor substrate-1; MAFBx=muscle atrophy f-box-1; MURF-1=muscle ring finger protein 1; NF κB= nucelar factor κB; IKK = inhibitor of nuclear factor κB kinase; IκB=inhibitor of nuclear factor κB; 4E-BP1= eukaryotic initiation factor (eIF) 4E binding protein 1; P70^S6K^ = 70-kDa ribosomal protein S6 kinase; mTOR= mammalian target of rapamycin; TNF-α= tumour necrosis factor-α

In health, net muscle synthesis is stimulated in the postprandial state while net muscle breakdown occurs between meals with muscle mass being maintained through balanced protein synthesis and breakdown [8]. Distinct metabolic pathways are involved in the synthesis and breakdown of muscle. Figure 1 provides an overview of these pathways. Muscle protein synthesis and not breakdown is more responsive to anabolic stimuli [9]. In healthy individuals, the anabolic effects of feeding occurs due to an increase in the synthetic rate of muscle protein synthesis of approximately 300 % with a concomitant 50 % decrease in the rate of protein breakdown [8, 10]. In healthy young men following an oral bolus of essential amino acids, there is a lag period of 45–90 min followed by an increase in the muscle protein synthetic response which continues for about 90 min then rapidly returns to baseline [8, 10, 11]. The duration and degree of the muscle protein synthetic response following protein ingestion is influenced by exercise, age and the dose and type of protein ingested and the anabolic/catabolic state of the individual [8]. It should be noted that other macronutrients have no additive anabolic effects and that the addition of carbohydrate to protein does not enhance muscle protein synthesis or attenuate muscle protein breakdown [8, 12].

In critical illness, loss of muscle mass results from an imbalance between muscle proteolysis and protein synthesis, with proteolysis overwhelming an inadequate synthetic response [13]. Proteolysis is mediated by several cellular signalling networks, but the predominant proteolytic pathway activated in models of muscle atrophy is the ubiquitin–proteasome system [14–16]. Two muscle-specific E3-ligases belonging to the ubiquitin–proteasome complex, muscle RING-finger 1 (MuRF1) and muscle atrophy F-box (MAFbx), have been identified as key regulators of proteasome-mediated protein breakdown [4, 17–19]. Forkhead box O (FOXO) are a family of transcriptional factors that plays a major role in muscle wasting primarily by increasing expression of MuRF-1 and MAFbx [20, 21]. FOXO is activated (dephosphorylated) by inflammation and sepsis.

Puthucheary and colleagues demonstrated a 17 % reduction in the rectus femoris cross-sectional area in critically ill patients after 10 days of mechanical ventilation [13]. Loss of muscle mass was greatest in those with multisystem failure and increased with increasing length of stay. In this study, the pattern of intracellular signalling demonstrated increased muscle breakdown and decreased synthesis. Wollersheim and colleagues investigated the dynamics of myosin degradation in patients requiring mechanical ventilation for at least 15 days [4]. These authors demonstrated decreased gene expression of the myosin heavy chain isoforms with significantly increased expression of MuRF-1, MAFbx and FOXO-1 mRNA. Constantin et al. reported similar findings; in addition, these authors reported widespread dephosphorylation (inactivation) of the proteins regulating translation initiation factor activation and protein synthesis (AKt1, mTOR, 4E-BP1) and increased expression of myostatin [22].

### Activation of muscle synthesis and the role of leucine

In skeletal muscle, the binding of insulin or insulin growth factor-1 (IGF1) activates the phosphoinositol-3 kinase/protein kinase B (PI3K/AKT) pathway inducing muscle synthesis by stimulating translation via mammalian target of rapamycin (mTOR) kinases [23]. In addition, IGF-1 suppresses MuRF1 transcription in part via the phosphatidyl-inositol 3 kinase/AKT pathway; Akt phosphorylates FOXO which is then sequestered in the cytoplasm preventing transcription of FOXO target genes [24]. mTOR exerts a critical role in mediating signal transduction necessary for mRNA translation initiation [8]. Rapamycin, a specific inhibitor of mTOR, signalling inhibits muscle protein synthesis in humans after an oral load of essential amino acids [25]. Key targets for mTOR activation include the 70-kDA ribosomal protein S6 kinase (p70^S6K^) and the eukaryotic initiation factor 4e-binding protein (4E-BP1) (see Fig. 1) [26]. Ingestion of protein in the form of free amino acids, milk protein or meat stimulates skeletal muscle protein synthesis at rest which increases further with exercise [27]. Postprandial muscle protein synthesis depends on the quantity and type of protein ingested. Activation of mTOR pathway is markedly increased following the ingestion of essential amino acids, particularly leucine. Activation of protein synthesis after acute resistance training is significantly reduced in the absence of essential amino acids [28, 29]. Essential amino acids have been described as ‘priming molecules’ whose phosphorylation of mTOR at Ser2448 is a prerequisite for further phosphorylation by Akt [26, 30]. Both leucine and AKt activate mTOR through phosphorylation of the Ser 2448 site [26, 31]. The activation of translation initiation by essential amino acids is independent of upstream IGF-1 signalling, with mTOR acting as a convergence point for the separate actions of amino acids and resistance exercise [26, 29]. Insulin increases muscle synthesis by multiple mechanisms including increased AKT/mTOR signalling and endothelial-dependent vasodilatation with an increase in nutritive flow [32].

Whey protein accounts for about 20 % and casein compromises about 80 % of total milk protein [33]. Whey protein is a rich source of leucine (14 %) and branched chain amino acids (26 %) [26]. The peak activation of muscle protein synthesis is reported to be proportional to the leucine content of the meal [34]. Whereas both casein and whey contain all the amino acids required to effectively stimulate muscle protein synthesis, whey has a considerably higher leucine concentration and has been demonstrated to have a greater postprandial muscle protein synthetic response than casein or soy [27, 35, 36]. Whey protein has been demonstrated to preserve muscle mass during intentional weight loss in obese adults [37]. Whey protein ingestion results in greater muscle protein synthesis than ingestion of its constituent amino acid content [38]. Bioactive peptides generated from whey protein have been demonstrated to stimulate the release of several gut hormones including cholecystokinin, peptide YY and the incretins glucose-dependent insulinotropic polypeptide 1 (GIP-1) and glucagon-like peptide (GLP-1) that potentiate insulin secretion [33, 39, 40]. In addition, these bioactive peptides inhibit dipeptidyl peptidase-4 (DPP4) preventing incretin degradation [33]. This may partly explain the greater insulin response following whey protein compared to casein [35]. In addition to its effects on protein synthesis, whey protein may limit autophagy by increasing activation of mTOR [41]. In a lipopolysaccharide (LPS) sepsis model, Tsutsumi et al. demonstrated that mice on a whey-based diet demonstrated improved survival with less mitochondrial autophagy and improved mitochondrial bioenergetics compared to mice on a casein-based diet [42]. Smith and colleagues demonstrated that dietary omega-3 fatty acid supplementation augments the hyperaminoacidemia–hyperinsulinemia-induced increase in the rate of protein synthesis [43, 44]. While the mechanism of this effect is not clear, the authors of this study demonstrated increased activation of the mTOR-p70^s6k^ signalling pathway without an effect on Akt signalling, suggesting increased activation at the level of mTOR.

### The kinetics of muscle protein synthesis

Optimal muscle protein synthesis requires a pulsatile increase in branch-chain amino acids (particularly leucine) with or without concomitant pulses in insulin levels. Pancreatic substrate clamp studies have demonstrated that insulin and branch-chain amino acids independently increase muscle synthesis with the effects of both being additive [45, 46]. Animal data demonstrates that muscle protein synthesis following a meal is rapid (within 30 min) and sustained for about 2 h but then declines toward baseline in parallel with the postprandial changes in circulating insulin and amino acids [34, 47]. Bohe and colleagues measured the latency and duration of the stimulation of human muscle protein synthesis during a continuous infusion of amino acids [48]. The rate of muscle protein synthesis increased after 30 min and reached a peak at 2 h rapidly returning to basal levels by 4 h despite continuous amino acid availability. In healthy individuals at rest, muscle protein synthesis displays a saturable effect which has been termed the ‘muscle full’ effect [11, 49]. The synthetic phase ends abruptly approximately 180 min after the initial food bolus despite ongoing provision of amino acids [10, 11]. Mitchell et al. have proposed a three-phase postprandial muscle synthetic response [11]. After the onset of essential aminoacidemia, a latent period exists providing time for adequate intracellular essential amino acid accumulation before muscle protein synthesis can be switched on. After this latent period, a transient stimulation in muscle protein synthesis lasting about 90 min occurs before the onset of the ‘muscle full’ state restores basal muscle protein synthesis despite sustained essential amino acid availability. West et al. demonstrated that myofibrillar protein synthesis after resistance exercise was significantly greater after the consumption of a single 25-g bolus dose of whey protein than when the whey protein was given as small pulsed drinks (ten 2.5-g drinks every 20 min) [50]. These concepts are supported by the study by Gazzaneo et al., who randomized neonatal pigs to receive a whey protein diet given as intermittent boluses (every 4 h) or as a continuous infusion for 24 h [51]. The authors then measured the degree of activation of the muscle anabolic pathways and the muscle protein synthetic rate in both groups. The serum levels of branch-chain amino acid and insulin levels spiked after each bolus whereas these levels remained flat in the continuously fed animals. Muscle protein synthesis 90 min after a bolus in the intermittently fed animals was twice that of the continuously fed animals. Phosphorylation of AKT, p70^S6K^ and 4E-BP1 was significantly increased in the bolus fed group while these biomarkers were at basal levels in the continuously fed animals.

The muscle synthetic response and the ‘muscle full’ effect are influenced by exercise, age, the type of protein ingested and the anabolic/catabolic state of the individual [8]. Decreased postabsorptive muscle protein synthesis termed ‘anabolic resistance’ is common with aging and may partly explain the sarcopenia of the elderly [8, 52]. Increased doses of protein and high-quality protein (whey) appear to overcome age-related anabolic resistance [53]. A 20-g dose of whey protein is required for the maximal myofibrillar synthetic rate in rested and exercised muscle of resistance-trained, young men [54] while 40 g is require in older adults [55]. Immobility [56–58], sepsis [59–61] and inflammation [62] result in anabolic resistance. Vary demonstrated that high dose leucine increases muscle protein synthesis and overcomes anabolic resistance in a murine sepsis model [63].

It is important to emphasize that the kinetics of muscle protein synthesis, the comparison of intermittent versus continuous supply of protein and the effect of whey- versus casein-based protein formula on protein synthesis have not been studied in critically ill patients. However, while the magnitude of the synthetic response may likely be blunted in critically ill patients as compared to healthy individuals, there is no physiological reason to believe that the stereotypic pattern of muscle synthesis noted in healthy individuals and animal models should not apply to the critically ill patient. In combat troops, protein dosing at a minimum of 20 g of high-quality protein every 4–5 h (during waking hours) has been recommended for optimal functional recovery [64]. While the optimal protein dose and dosing strategy in critically ill patients is unknown, I suggest that an approach similar to that of combat troops may limit the loss of muscle in these patients.

### Clinical studies fail to demonstrate improved outcome with more protein (provided continuously)

Four randomized controlled trials have been performed comparing permissive underfeeding to full feeding or standard feeding to ‘PepUp’ feeding [65–68]. None of these trials demonstrated an improvement in any clinical outcome by providing more calories and more protein. The largest of these trials, the EDEN study randomized patients (*n* = 1000) with acute lung injury to receive either trophic feeding at 20 kcal/h (which is about 7 cal/kg/day) or full feeding at 25–30 kcal/kg/day for the first 6 days (the average protein dose received in each group was not reported!) [65]. After day 6, all patients who were still receiving mechanical ventilation received the full feeding protocol. There was no difference in the number of ventilator-free days (primary outcome), 60-day mortality and other secondary end-points between groups. Follow-up of these patients showed no difference in physical, psychological and cognitive function as well as quality of life at 12 months [69, 70]. In the study by Arabi et al., hospital mortality was lower in the permissive underfeeding group than in the target feeding group (30.0 % vs 42.5 %; RR 0.71; 95 % CI: 0.50, 0.99; *P* = 0.04) [66]. In all of these studies, patients were fed enterally with a continuous supply of amino acids. The Early Parenteral Nutrition Completing Enteral Nutrition in Adult Critically Ill Patients (EPaNIC) was a prospective RCT that compared early with late initiation of parenteral nutrition in ICU patients unable to tolerate adequate enteral nutrition [71]. In a post hoc analysis of this study, Casear et al. demonstrated a strong association between increasing cumulative protein intake (given as a continuous infusion) with a lower likelihood of an earlier alive-discharge from the ICU [72]. I postulate that the negative outcomes of these studies are related to the fact that the increased dose of protein/amino acids were given as a continuous infusion. It is noteworthy that in the study of Puthucheary and colleagues (referenced above) a higher protein delivery during the first week of critical illness was associated with greater muscle wasting [13].

### Intermittent vs continuous feeding

No species eats continuously (day and night) and such an evolutionary design would seem absurd. The alimentary tract and metabolic pathways of humans appear designed for intermittent ingestion of nutrients a few times a day. Humans have evolved, as intermittent meal eaters are not adapted to a continuous inflow of nutrients; normal physiology appears to be altered when this approach is adopted. However, continuous enteral feeding of critically ill patients appears to be the standard of care around the world [73]; such an approach is clearly unphysiological and likely to be associated with significant complications. Rapid syringe bolus feeding was the norm prior to the introduction of continuous infusion pumps. Rapid bolus feeding was associated with sudden gastric distension and a high incidence of nausea and vomiting. Continuous feeding with an infusion pump was associated with less intolerance and soon become considered the standard of care. However, when the ‘bolus’ of enteral feed is given over a longer period of time (20–40 min), the incidence of nausea and vomiting is not increased [74]; this method of feeding is best referred to as intermittent feeding.

In addition to adversely affecting protein synthesis, continuous enteral feeding has other adverse consequences (see Table 1). The gastrointestinal tract is an important endocrine organ with dozens of regulatory peptides being produced by specialized endocrine cells within the gastrointestinal mucosa. These hormones serve complex roles regulating gastrointestinal motility, gall bladder contraction, pancreatic function and nutrient absorption [75]. The majority of these hormones are secreted within minutes of nutrient ingestion and rise transiently in the circulation with levels falling back to basal levels after termination of feeding. This entero-hormonal response to nutrient ingestion is almost completely abolished following continuous tube feeding. The incretins, glucose-dependent insulinotropic polypeptide (GIP) and glucagon-like peptide-1 (GLP-1) play an important role in the coordinated response to the incoming carbohydrate load [75]. Both these hormones potentiate insulin secretion from the islet-β cell in a glucose-dependent manner and account for up to 70 % of insulin release [76, 77]. Stoll et al. studied the kinetics of incretin release and gastrointestinal function in neonatal pigs who received continuous or intermittent enteral feeding [78]. In this study, blood GIP and GLP-1 levels as well as insulin receptor phosphorylation in liver and muscle were significantly reduced in the continuously fed as compared to the intermittently fed animals. Furthermore, ileal mass and villus height were significantly less while hepatic steatosis and hepatic inflammation were significantly greater in the continuously fed animals. Similarly, Shulman et al. compared bolus versus continuous tube feedings on small-intestinal growth and development in newborn pigs [79]. In this study, small-intestinal mucosal weight, ileal protein mass and mucosal enzymatic activity were significantly less in the continuously fed animals. In a randomized crossover study, Chowdhury et al. compared bolus with continuous nasogastric feeding in healthy human adults [80]. In this study, bolus feeding led to a significant increase in mesenteric artery blood flow and an increase in the concentration of insulin and peptide YY; these variables remained virtually flat in the continuously fed group. Furthermore, the mean blood glucose concentration was significantly lower in the bolus fed group over the 4-h study period (*P* < 0.0001). It should be noted that GLP-1 results in skeletal muscle microvascular recruitment with increased blood flow [81, 82] and nutrient delivery and this may play a role in the coordinated postprandial muscle synthetic response as already discussed. Recent studies have demonstrated that there are GLP-1 receptors on many organs and tissues including the kidney, brain and heart and that GLP-1 has neuro-protective, cardio-protective, reno-protective and anti-inflammatory properties [77, 83, 84]. It is possible that the blunted release of GLP-1 may contribute to organ dysfunction in continuously fed critically ill patients. Intermittent oral feeding results in pulsatile cholecystokinin (CCK) release with gall bladder emptying whereas continuous enteral feeding results in a blunted CCK response and an enlarged non-contractile gall bladder [85, 86]. Impaired release of bile may result in impaired lipid absorption and diarrhoea commonly noted in the critically ill. Furthermore, impaired gall bladder contractility may account for the high incidence of acalculous cholecystitis which occurs in this patient population [87].

**Table 1:** Potential harm associated with continuous tube feed

Organ system	Potential adverse effect
Muscle	Decreased skeletal muscle synthesis
Endocrine	Decreased secretion of GIP, GLP-1, peptide YY and CCK
	Decreased insulin release
	Insulin resistance
	Hyperglycemia
Gastrointestinal	Hepatic steatosis
	Hepatic inflammation
	Enlarged non-contractile gall bladder
	Impaired lipid absorption
	Small bowel atrophy
	Impaired small bowel function
	Decreased mesenteric blood flow
Other	Multi-organ dysfunction syndrome

*GIP* glucose-dependent insulinotropic polypeptide, *GLP-1* glucagon-like peptide-1, *CCK* cholecystokinin

Based on these data, I suggest that it is illogical to feed patients with a continuous infusion of enteral nutrition (a parenteral infusion would be more illogical). A limited number of studies have been performed comparing continuous to intermittent enteral nutrition [74, 88, 89]. While these studies did not evaluate patient centered outcomes such as mortality, ventilator-free days, muscle function or metabolic parameters they demonstrated that this approach is both safe and feasible. MacLeod and colleagues randomized 164 trauma patients to an intermittent feeding regimen (one-sixth of daily needs infused every 4 h) or a continuous feeding regimen [74]. The intermittent feed was delivered via an enteral feeding pump over a 30- to 60-min period of time. These investigators reported no difference in the complication rate between groups (diarrhoea and pneumonia); however, the caloric goal was achieved earlier in the intermittently fed patients. We currently have experience with feeding over 300 patients by the intermittent method. This feeding technique has been very well tolerated by our patients with no evidence of an increase in the risk of aspiration or diarrhoea. Compared to historical controls, our data suggests that glycemic control improves with intermittent feeding (significantly lower percentage of patients with blood glucose >180 mg/dl). Due to the improved gastrointestinal tolerance, improved glycemic control, perceived clinical benefits and ease of administration, intermittent bolus feeding is preferred over continuous feeding by our ICU nursing staff and dieticians. It should be noted that the intermittent boluses are given using an enteral feeding pump over a 20- to 40-min period. While the optimal amount of calories and protein that should be given with the intermittent approach is unknown, we target 20–25 cal/kg/day divided into 6 aliquots given every 4 h. We use a whey-based formula (with omega-3 fatty acids) with a caloric density of 1.2 calories/ml with an average target of 1800 calories (250 ml q 4 hr). The protocol for escalation of intermittent feeds is provided in Table 2.

**Table 2:** Intermittent feeding schedule

Time (h)	Volume (ml)	Duration of infusion (min)
0	100	20
4	150	20
8	150	20
12	200	30
16	200	30
20	250	40
24	Target	

It is my opinion that continuous enteral nutrition is unphysiological, limits preservation of muscle mass and is associated with adverse effects on glucose and lipid metabolism and that this approach to nutritional support should be abandoned. Experimental and clinical studies have shown that ‘mechanical silencing’ of skeletal muscle plays a major role in CIM [56, 90]. Loss of muscle mass and function can be attenuated by early mechanical loading [91, 92], supporting early physical therapy in immobilized patients [93–95]. It is therefore my belief that optimal nutritional support provided by intermittent feeding of a whey-based enteral formula combined with early physical therapy may attenuate CIM.

## Conclusions

In conclusion, I believe that critically ill patients should be fed intermittently with a whey-based formula which contains omega-3 fatty acids. Such an approach is likely to limit muscle atrophy and promote metabolic stability. Continuous tube feeding is unphysiological and likely harmful and should be abandoned. Large randomized controlled trials are urgently required to demonstrate the clinical benefits of an intermittent feeding strategy.

## Abbreviations

CCK: Cholecystokinin
CIM: Critical illness myopathy
FOXO: Forkhead box O
GIP: Glucose-dependent insulinotropic polypeptide
GLP-1: Glucagon-like peptide-1
GRV: Gastric residual volumes
IGF1: Insulin growth factor-1
ICUAW: Intensive care unit-acquired weakness
LPS: Lipopolysaccharide
MuRF1: Muscle RING-finger 1
MAFbx: Muscle atrophy F-box
mTOR: Mammalian target of rapamycin
TNF-α: Tumour necrosis factor-α
TGF-β: Transforming growth factor-β
PI3K/AKT: Phosphoinositol-3 kinase/protein kinase B (PI3K/AKT)
